# *Notes from the Field:* Clinical *Klebsiella pneumoniae* Isolate with Three Carbapenem Resistance Genes Associated with Urology Procedures — King County, Washington, 2018

**DOI:** 10.15585/mmwr.mm6830a4

**Published:** 2019-08-02

**Authors:** Kirsten Vannice, Eileen Benoliel, Kelly Kauber, Claire Brostrom-Smith, Patricia Montgomery, Meagan Kay, Maroya Walters, Michael Tran, Marisa D’Angeli, Jeff Duchin

**Affiliations:** ^1^Epidemic Intelligence Service, CDC; ^2^Public Health — Seattle & King County, Seattle, Washington; ^3^Washington State Department of Health, ^4^Division of Healthcare Quality Promotion, National Center for Emerging and Zoonotic Infectious Diseases, CDC.

On December 31, 2018, Public Health — Seattle & King County (PHSKC) was notified by the Antibiotic Resistance Laboratory Network regarding a carbapenem-resistant *Klebsiella pneumoniae* (CR-Kp) isolate cultured from the urinary tract in a man aged 65 years. The specimen was collected on December 17, 2018. It tested positive for carbapenemase activity by the modified carbapenem inactivation method and positive for genes encoding the carbapenemases New Delhi metallo-beta-lactamase, Verona integron-encoded metallo-beta-lactamase, and OXA-48–type beta-lactamase, by polymerase chain reaction. Antimicrobial susceptibility testing by broth microdilution showed resistance to 15 antibiotics tested* but low minimum inhibitory concentrations (MIC) to colistin (MIC ≤0.25) and tigecycline (MIC = 1). CDC recommends a public health response when organisms with emerging forms of antibiotic resistance, such as the metallo-beta-lactamases this isolate harbored, are identified^†^ because such organisms are often difficult to treat and have the potential to spread rapidly in health care settings (*1*).

Since September 2017, the patient had been treated at a local outpatient urology clinic (facility A) in King County, Washington, for lower urinary tract symptoms that included urinary retention and benign prostatic hyperplasia. Procedures at facility A before identification of CR-Kp included cystoscopy, urinary catheter placement, and computed tomography scan of the urinary tract. On November 1, 2018, he underwent outpatient urodynamic studies at a hospital in Punjab, India (facility B), where urinary and rectal catheters were placed for cystometric and pressure flow studies; he did not undergo cystoscopy and was not hospitalized. Lower urinary tract symptoms persisted, and upon the patient’s return to the United States, a urinalysis at facility A on December 17 revealed an elevated white blood cell count and presence of leukocyte esterase, which led to the urine culture that identified CR-Kp. The patient’s chronic symptoms and signs remained unchanged, and he was not treated at the time of CR-Kp identification per standard guidelines (*2*,*3*). Urinary and rectal specimens to screen for carbapenem-resistant Enterobacteriaceae (CRE) were collected on February 18, 2019; both specimens were negative by polymerase chain reaction (rectal swab) and culture (urine).

After identification of CR-Kp, education was provided to the patient and his family about preventing spread of the organism in the home and sharing this medical history with future medical providers (*4*). Facility A flagged the patient record for contact precautions. Staff members from PHSKC and the Washington Department of Health inspected facility A to ensure infection control procedures were in place. CRE identifications are notifiable conditions in Washington (*2*), and no other patients with matching CR-Kp strains have been identified from facility A. PHSKC recommended that facility A maintain contact precautions for the patient, pending results of rescreening for CRE 6 months after the positive culture.

This case highlights two important considerations for preventing antibiotic resistance spread. First, when responding to emerging antibiotic resistance, health departments should consider on-site standardized infection control assessments^§^ in outpatient settings associated with an elevated risk for transmission, such as those that perform procedures involving the digestive tract, urogenital tract, and wounds. Second, providers should obtain thorough travel histories for any medical care received outside of the United States during the previous 6 months (*5*). Patients who had overnight hospital stays or underwent outpatient medical procedures abroad should be considered at risk for colonization or infection with CRE. To quickly identify and prevent CRE transmission, providers should consider CRE screening for patients who have undergone medical procedures abroad and who will be hospitalized or undergo invasive procedures in the United States. Carbapenem-resistant organisms from patients with this history should be tested for carbapenemases. Carbapenemase testing for CRE and carbapenem-resistant *Pseudomonas aeruginosa* and screening for carbapenemases in suspected isolates may be requested by state health departments through the Antibiotic Resistance Laboratory Network.^¶^

## Footnotes

* amikacin, aztreonam, cefepime, ciprofloxacin, doripenem, doxycycline, ertapenem, gentamicin, imipenem, levofloxacin, meropenem, minocycline, piperacillin/tazobactam, tobramycin, and trimetho/sulfa.

† https://www.cdc.gov/hai/containment/guidelines.html.

§
https://www.cdc.gov/infectioncontrol/pdf/icar/outpatient.pdf .

¶ https://www.cdc.gov/drugresistance/solutions-initiative/ar-lab-network.html.
